# The functionality of the cysteinyl leukotriene receptor 1 (CysLTR1) in the lung by metabolomics analysis of bronchoalveolar lavage fluid

**DOI:** 10.1007/s11306-026-02491-9

**Published:** 2026-07-08

**Authors:** Wilson B. Adeosun, Sibongiseni K. L. Poswayo, Suraj P. Parihar, Du Toit Loots

**Affiliations:** 1 Biomedical and Molecular Metabolism (BioMMet), North‒West University, Hoffman Street, Potchefstroom, 2531 South Africa; 2 Division of Medical Microbiology, Institute of Infectious Diseases and Molecular Medicine (IDM), Faculty of Health Sciences, University of Cape Town, Cape Town, South Africa; 3 Centre for Infectious Disease Research in Africa (CIDRI- Africa), Faculty of Health Sciences, University of Cape Town, Cape Town, South Africa

**Keywords:** Cysteinyl leukotriene receptor 1, Bronchoalveolar lavage fluid, Metabolomics

## Abstract

**Introduction:**

The cysteinyl leukotriene receptor 1 (CysLTR1) is known as a potent lipid mediator with a well-established role in inflammatory regulation and lung disease. While its involvement in immune cell recruitment has been previously reported, its broader impact on pulmonary metabolism remains poorly understood.

**Objectives:**

The study aims to investigate the metabolic consequences of a CysLTR1 deletion in mice to elucidate its role in pulmonary metabolic homeostasis.

**Methods:**

Bronchoalveolar lavage fluid (BALF) was collected from CysLTR1 knockout (KO) and wild-type (WT) mice (*n* = 4 per group), and analysed using standardized untargeted gas chromatography–time-of-flight mass spectrometry (GC-TOFMS) metabolomics.

**Results:**

Metabolomics analyses of the BALF collected from the CysLTR1 KO mice presented significantly reduced levels of glucose, glucosamine, and glyceric acid, indicating the role of the CysLTR in lung glucose uptake and consequently lung glycolysis and gluconeogenesis. This is further supported by reductions in myo-inositol and D-chiro-inositol, also supporting previous findings that this occurs due to insulin resistance. Consequential disruption of various glucose-dependent pathways, including the pentose phosphate pathway (reduced gluconic acid, sedoheptulose and xylose) and purine metabolism (reduced 1-methylinosine) indicates a consequential altered nucleotide turnover, and the significantly reduced concentrations of butanoic acid, decan-2-ol, and 1-hexadecanol, indicate changes to fatty acid metabolism in the lung, as a compensatory response to the initial glucose deficiency induced by the CysLTR1 KO. Lastly, the changes to mandelic acid, glutaric acid, tricarballylic acid, and decan-2-ol, furthermore, indicate the role of CysLTR1 in the composition/metabolism of the microbiome.

**Conclusion:**

This study expands our knowledge on the role of CysLTR1 beyond its role in immune regulation, which may contribute to a better understanding of CysLTR1 associated lung diseases and in the development of improved therapeutic strategies.

**Supplementary Information:**

The online version contains supplementary material available at https://doi.org/10.1007/s11306-026-02491-9.

## Introduction

Leukotrienes (LTs) and cysteinyl leukotrienes (CysLTs; LTC4, LTD4 and LTE4) are lipid mediators derived from arachidonic acid via the 5-lipoxygenase pathway, and function as regulators of inflammation, vascular permeability, and smooth muscle contraction (Kanaoka & Boyce, 2004). Leukotrienes, especially leukotriene B4 (LTB4), regulate how macrophages respond to antigens, and are associated with inflammation. This phenomenon is important in the progression of various diseases, including atherosclerosis (Sánchez-Galán et al., 2009), obesity and type 2 diabetes (Filgueiras et al., 2015; Mothe-Satney et al., 2012). Considering the latter, the binding of LTB4 to its receptor results in various inflammatory responses, leading to insulin resistance. Interestingly, elevated concentrations of LTB4 in type 2 diabetes patients have also been shown to be correlated with cardiovascular autonomic dysfunction (Neves et al., 2020).

The cysteinyl leukotrienes function via the G protein-coupled receptors - CysLTR1, CysLTR2, and CysLTR3, and are best described for their role in various allergic and asthmatic responses and inflammation. For example, the CysLTs regulate the production of proinflammatory cytokines and adipokines, including interleukin 6 (IL-6), monocyte chemotactic protein 1, tumor necrosis factor-α (TNF-α), nuclear factor kappa B (NF-κB), and macrophage inflammatory protein 1 (Coffey et al., 2015; Filgueiras et al., 2015). Furthermore, CysLTR1 mediates inflammation during infection, and it has been demonstrated that mice lacking CysLTR1 have reduced granuloma, hepatic fibrosis, and liver enzyme release, which in turn is associated with an increased anti-inflammatory cytokine production. Furthermore, treating mice with montelukast (a CysLTR1 antagonist) in combination with praziquantel, resulted in a reduced cellular infiltration into the liver and a reduced egg burden during chronic *Schistosoma mansoni* infection, and a combination therapy of CysLTR1 inhibition and praziquantel, serves as a prophylactic treatment approach (Mosala et al., 2024). Eosinophils, mast cells, and chemosensory epithelial airway tuft cells, are also reportedly activated by CysLTs, leading to the type-2 immune responses, mucus secretion and exacerbation of pulmonary illnesses (Liu et al., 2025; Salimi et al., 2017). More recently, CysLTR1 inhibition has been shown to modulate intracellular glucose levels in retinal endothelial cells, pericytes and ARPE-19 cells, indicating a possible role in cellular glucose metabolism (Koller et al., 2020; Koller et al., 2025).

Bronchoalveolar lavage fluid (BALF) is an important biofluid used for researching changes to the lung microenvironment. The procedure involves washing the lower respiratory tract (bronchoalveolar space) with saline solution, which is then recovered. The solute content is composed mostly of alveolar macrophages, lymphocytes, neutrophils, proteins and primary lung metabolites (Kalidhindi et al., 2021; Sabounchi-Schütt et al., 2003). A metabolomics analysis of BALF would subsequently provide a detailed snapshot of the metabolic changes in the the lung due to a perturbation. This study subsequently investigated the role of CysLTR1 on the lung by comparing the metabolic changes associated with a CysLTR1 KO mouse model with that of a WT control, using GC-TOFMS metabolomics analysis of BALF samples.

## Materials and methods

### Reagents

Deionized water from a Millipore Milli-Q purification system was used throughout the study. Optima-grade acetonitrile was obtained from Fisher Scientific (Pittsburgh, USA). Unless otherwise stated, all other reagents and organic solvents used in this investigation were sourced from Sigma‒Aldrich (St. Louis, MO, USA).

### Samples

CysLTR1-deficient (Cysltr1−/−) mice were generated by breeding heterozygous (Cysltr1−/+) animals on a C57BL/6 background, generously provided by Dr. Frank Austen (Harvard Medical School). Genotypic confirmation was performed by PCR-based analysis of genomic DNA extracted from ear biopsies, yielding the expected amplicon sizes for Cysltr1−/− (333 bp) and wild-type Cysltr1+/+) (284 bp) mice, allowing unambiguous discrimination between genotypes. Functional validation of gene deletion was further confirmed at the transcript level by quantitative PCR analysis of CysLTR1 mRNA expression in multiple tissues, including thymus, spleen, liver, and lung, normalized to the HPRT housekeeping gene, demonstrating near-complete absence of CysLTR1 transcripts in knockout mice. Age- and sex-matched male mice (8–12 weeks old; *n* = 4 per group) were used. All animals were housed under specified pathogen-free (SPF) conditions at the Animal Research Facility, Faculty of Health Sciences, University of Cape Town. Mice were acclimated for 7 days prior to experimentation and individually housed in transparent individual ventilated cages (IVCs) maintained at 22 ± 2 °C and 50–60% relative humidity on a 12 h light/dark cycle, with standard irradiated chow and water provided ad libitum. Bedding consisted of autoclaved wood shavings and shredded paper, supplemented with perplex nesting material to promote environmental enrichment and burrowing behaviour; cages were changed weekly by trained personnel using low-stress handling techniques. For BALF collection, mice were terminally anesthetized, a catheter was inserted into the trachea and secured, and a syringe containing 1 mL of sterile saline was gently instilled and aspirated while massaging the thorax, as previously described (Thawer et al., 2016). Approximately 500–800 µL of BALF was recovered per mouse, transferred to 2 mL cryotubes, snap-frozen in liquid nitrogen, and stored at − 80 °C until analysis. All experimental procedures were conducted in accordance with the South African National Standard for the Care and Use of Animals for Scientific Purposes (SANS 10386:2008) and were approved by the Animal Research Ethics Committee of the University of Cape Town (AREC Permit No. 022/024).

### Sample preparation and GC-MS analysis

A 100 µL volume of each BALF sample, was centrifuged at 10, 000 rpm for 10 min at room temperature, after the addition of 50 µL of a 3-phenylbutyric acid (Sigma‒Aldrich) internal standard (50ppm; retention time 16.89 min) and 300 µL of acetonitrile. The supernatant was subsequently evaporated to dryness before derivatization. To monitor analytical reproducibility and ensure data quality, pooled QC samples were prepared by combining equal volume aliquots from each experimental BALF sample included in the study prior to extraction. The pooled QC sample was further aliquoted and treated as a biological sample, undergoing the same sample extraction, and derivatisation steps. Extraction blanks, went through the sample preparation, extraction and derivatisation steps, but did not contain any biological sample. The dried metabolic extract was oximated using 50 µL of methoxyamine hydrochloride in pyridine (15 mg/mL) (Merck, Darmstadt, Germany) at 50 °C for 1 h, and then silylated using 50 µL of N, O-bis (trimethylsilyl) trifluoroacetamide (BSTFA) with 1% trimethyl chlorosilane (TMCS) (Sigma‒Aldrich, St. Louis, MO, USA) at 60 °C for 1 h. The derivatized sample extracts were transferred to 2 mL glass vials containing 250 µL glass inserts.

One microliter of each sample extract was injected (1:1 split ratio) into a Pegasus BT GC-TOFMS instrument (Leco Corporation, St. Joseph, MI, USA), equipped with an Agilent 7890 A gas chromatograph (Agilent, Atlanta, GA, USA) coupled to a time‒of-flight mass spectrometer (TOFMS) (Leco Corporation, St. Joseph, MI, USA). Separation was achieved via an Rxi-5-MS column (29.690 m, 0.25 mm internal diameter and 0.25 μm film thickness) (Restch GmbH & Co. KG, Haan, Germany). The front inlet temperature was held at a constant 270 °C, the transfer line temperature at a constant 250 °C, and the ion source temperature at a constant 200 °C for the entire run. The initial GC oven temperature was set at 70 °C for 1 min, followed by an increase of 5 °C/min to a final temperature of 320 °C, at which it was held for 3 min. The detector acquisition delay for each run was 420 s and was offset with a filament bias of − 70 eV. Spectra were collected from between 50 and 950 m/z at an acquisition rate of 20 spectra per second.

The extracted BALF samples were randomly injected, ensuring that any residual analytical variation was equally distributed among the groups, thereby minimizing potential bias. The prepared QCs, extraction blanks, and a FAMEs standard, were injected 3 times at the beginning, middle and end of the sample batch.

### Data processing

Mass spectral deconvolution and peak identification were performed via Leco Corporation’s ChromaTOF software (version 4.71), at a signal-to-noise ratio of 30, with a minimum of three apex peaks. Peak annotations were performed by comparing the mass fragment patterns of the analyte to those of the commercially purchased National Institute of Standards and Technology (NIST) mass spectra library and in-house libraries generated from previously injected standards, using a similarity threshold of at least 70%. In accordance with the Metabolomics Standards Initiative (MSI) reporting framework (Sumner et al., 2007), all reported metabolites are classified as MSI Level 2 identifications. To eliminate the effects of retention time shifts and create a data matrix containing the relative abundances of all the compounds present in all the samples, peaks with identical mass spectra and retention times were aligned using ChromaTOF’s “Statistical Compare” function, resulting in a dataset containing 936 compounds. The dataset was meticulously cleaned to eliminate compounds without matching mass spectral entries in the libraries, and other artifact compounds of no biological relevance. The raw metabolite peak intensities of the remaining compounds were subsequently normalized relative to the internal standard, 3-phenylbutyric acid, which was added at a fixed concentration to all samples prior to extraction. The relative metabolite concentrations were determined using the ratio of the metabolite peak area to the peak area of the internal standard according to the following equation:

$$\:{C}_{metabolite}=\frac{{A}_{metabolite}}{{A}_{IS}}\times\:F$$

where $\:{C}_{metabolite}$ is the relative concentration of the metabolite, $\:{A}_{metabolite}$ the metabolite peak area, $\:{A}_{IS}$ the peak area of the internal standard, and * F* the correction factor (concentration of internal standard in each sample).

Standard metabolomics data clean-up procedures were followed as previously described (Gromski et al., 2014; Sullivan & Feinn, 2012). Briefly, compounds with more than 50% zero values within the groups were excluded from further analysis. Batch correction (using quantile equating) and a coefficient of variation (CV) filter (retaining compounds with CV ≤ 50%) were applied using the QC samples. Zero values detected for a compound were replaced with half the smallest detected value to reflect the lower detection limit (Luier & Loots, 2016). A total of 201 annotated compounds across the quality control (QT), WT, and KO samples were retained for downstream statistical analyses.

### Statistical analysis

Normalized data were subsequently log-transformed to improve normality prior to downstream univariate and multivariate statistical analyses, including t-test, log2-fold change (log2FC), and effect size analyses, to determine which metabolites contributed most significantly to the observed changes between the sample groups. P-values were adjusted for multiple test correction using FDR correction, and all reported significant values in the Results section reflect FDR-adjusted p-values. Effect sizes were calculated using Cohen’s d, followed by principal component analysis (PCA) to determine the compounds contributing most to the observed variability and their relationships with the groups. All data analyses were performed using MetaboAnalyst 6.0 (Pang et al., 2024).

## Results

### Data quality evaluation

**Fig. 1:**
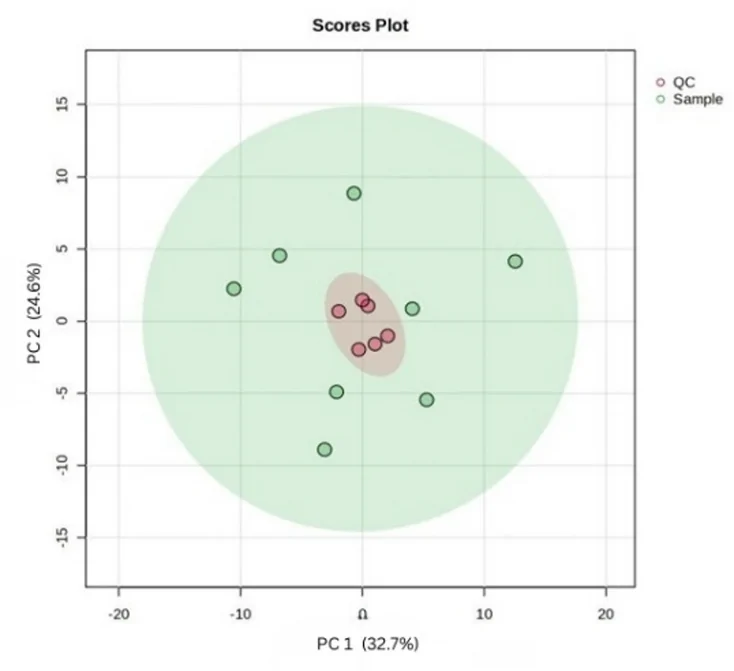
Principal component analysis score plot generated using the normalized relative concentrations of 201 annotated compounds detected, showing distinct clustering of quality control samples (QCs) and separation between BALF experimental groups (samples)

As indicated in Fig. 1, the consistent grouping of the QC samples within that of the experimental samples supports the reliability of the data and suggests that instrument drift or analytical variability is absent. These observations affirm the quality and reproducibility of the dataset, validating its suitability for downstream multivariate and univariate statistical analyses.

### Chemometric analysis

**Fig. 2:**
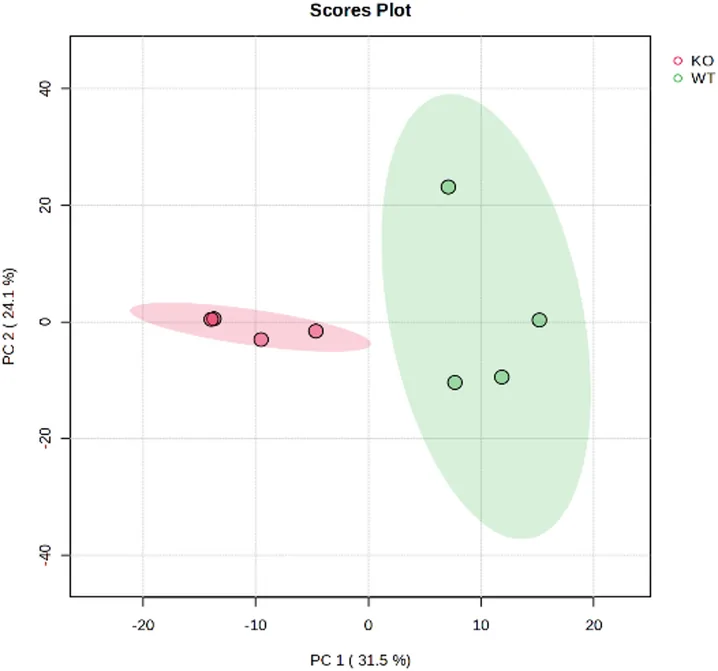
shows the PCA generated using the normalized relative concentrations of the 201 annotated compounds detected across the CysLTR1 KO and WT sample groups. The x-axis (PC1) explains 31.5% of the total variance, whereas the y-axis (PC2) accounts for 24.1%

Figure 2 Principal component analysis score plot generated using the normalized relative concentrations of 201 annotated compounds from the CysLT1 KO and WT sample groups. The percentages in parenthesis represent the proportion of variation explained in the observed data by the specific principal component (PC).

The clear separation between these groups along both PC1 and PC2 indicates significant metabolic differences between the CysLTR1 KO and WT groups.

### Statistics and metabolite marker selection

**Fig. 3:**
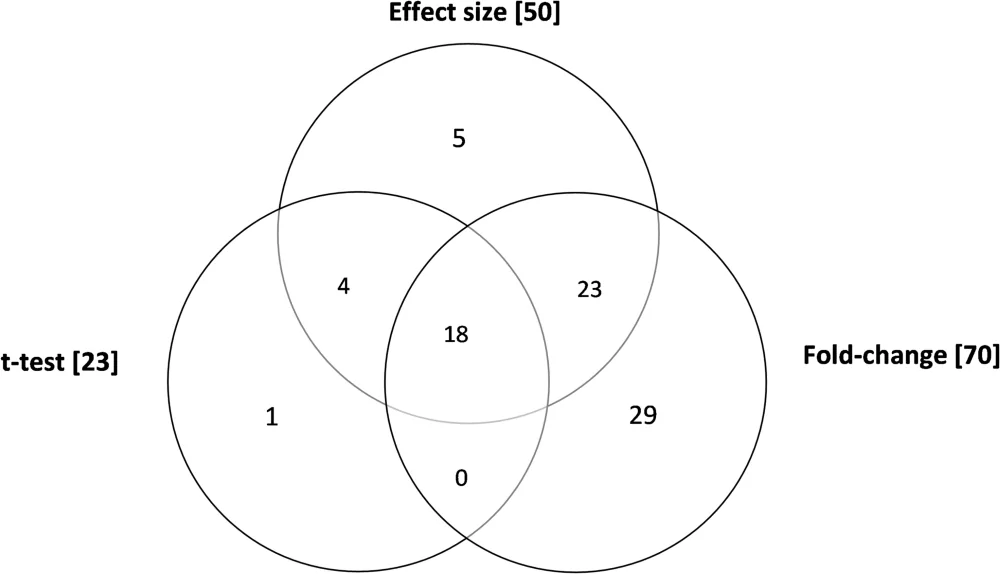
Venn diagram indicating the selection of the 18 metabolite markers contributing most significantly to the differences observed when comparing the CysLTR1-KO and WT BALF sample groups via a multistatistical approach. The selection criteria included metabolites having a t-test p-value < 0.05, a Log2FC of threshold 0.5, and an effect size ≥ 0.8

Of the aforementioned 201 metabolites in the final data set, 23 metabolites showed a significant t-test p-value of < 0.05, 70 metabolites a log2FC threshold beyond 0.5, and 50 metabolites a Cohen’s d ≥ 0.8, when comparing the groups. The 18 metabolite markers that best describe the variance between CysLTR1 KO-deficient and WT BALF samples were selected via the multistatistical selection approach as illustrated in the Venn diagram in Fig. 3.

Table 1 presents the metabolites that most significantly differ when comparing the CysLTR1 KO and WT BALF groups.

**Table 1:** Metabolites with most significance when comparing the CysLTR1 KO and WT BALF sample groups

Metabolite (PubChem ID)	Average relative concentration (ng/mL)/(standard deviation) Wild type mice CysLTR1 KO mice		Log2FC (threshold 0.5)	Cohens (effect size: d-value ≥ 0.8)	t-test *p*-value < 0.05
Tricarballylic acid (14925)	2.650 (1.994)	0.792 (0.100)↓	−4.029	2.911	0.001
Glucosamine (739)	9,707 (10.121)	5,825 (8.381)↓	−2.45	1.958	0.026
Decan-2-ol (2737541)	1.117 (0.987)	0.120 (0.269)↓	−2.435	1.945	0.027
D-Chiro-Inositol (16216949)	125.524 (60.474)	56.153 (14.225)↓	−1.639	2.558	0.013
Glucose (5793)	2.118 (1.647)	0.780 (0.593)↓	−1.440	0.824	0.036
Mandelic acid (22943025)	52.300 (18.357)	38.746 (37.000)↓	−1.366	3.362	0.002
D-xylose (135191)	23.682 (19.270)	9.937 (0.002)↓	−1.713	1.307	0.033
Glutaric acid (90037731)	37.290 (17.780)	20.310 (5.905)↓	−1.343	2.186	0.008
Sedoheptulose (5459879)	19.677 (6.298)	12.138 (4.172)↓	−1.191	2.803	0.002
Glyceric acid (752)	55.715 (12.841)	31.324 (7.776)↓	−1.174	2.939	0.002
Gluconic acid (10690)	222.814 (134.281)	155.146 (80.397)↓	−1.435	1.624	0.022
1-Hexadecanol (2682)	38.672 (19.981)	22.849 (9.901)↓	−1.273	1.758	0.011
(R*,S*)−2,3 Dihydroxy butanoic acid (250402)	8.782 (3.632)	5.921 (1.384)↓	−1.101	2.605	0.006
Butanoic acid (16213394)	325.566 (117.085)	207.031 (36.553)↓	−0.981	2.737	0.007
Myo-inositol (440388)	1912.119 (865.363)	1398.837 (441.522)↓	−0.932	1.676	0.037
Galactose (441035)	25632.153 (6700.214)	20646.841 (2718.550)↓	−0.632	2.766	0.003

## Discussion

**Fig. 4:**
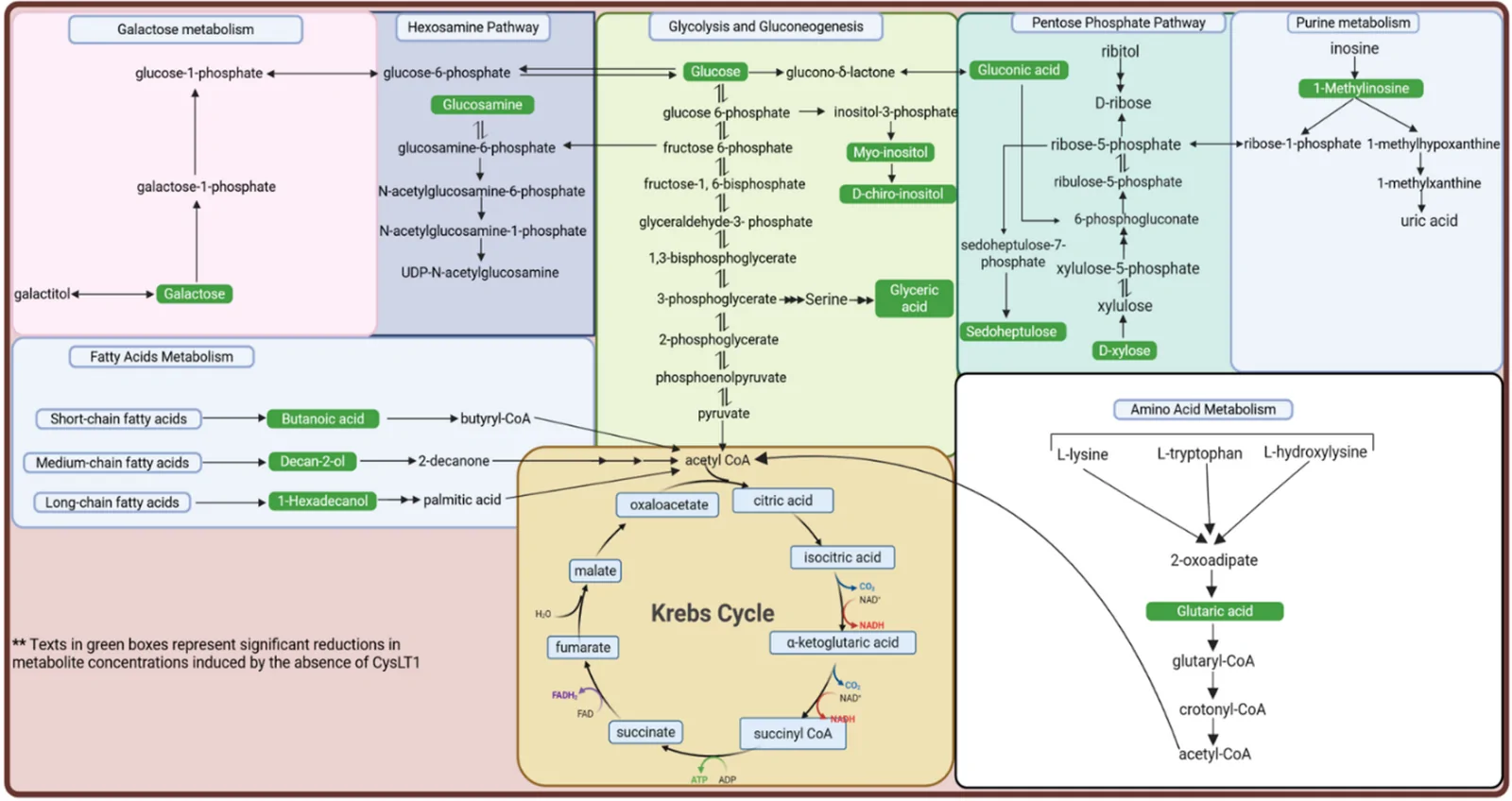
Schematic summary of the most significantly altered metabolites (highlighted in green) shown to be reduced in the lung BALF samples due to the absence of CysLTR1

Figure 4 shows a schematic summary of those metabolites most significantly changed in the mice BALF samples collected from the lungs, due to the absence of CysLTR1 (highlighted in green), when compared to the WT mice. The altered pathways in the CysLTR1 KO included changes to glycolysis, the pentose phosphate pathway, purine metabolism, amino acid metabolism, fatty acid metabolism, galactose metabolism, and the hexosamine biosynthesis pathway.

The metabolomic analysis of biofluids such as serum, plasma, urine, cerebrospinal fluid, and BALF is a powerful tool for understanding the physiological and pathological states of disease conditions and, in this case, that of the lung (Evans et al., 2014; Stringer et al., 2016). In clinical research, providing a comprehensive snapshot of metabolic alterations in a biological system/sample/tissue, is important for identifying disease biomarkers, elucidating changes to biochemical pathways, and monitoring therapeutic responses (Dar et al., 2023; Perakakis et al., 2018). The results of the present investigation provide useful insights into the biochemical and physiological changes associated with CysLTR1, further highlighting the potential role of CysLTR1 in pulmonary physiology and disease mechanisms, as revealed by changes in the metabolic pathways influenced by the absence of CysLTR1 in the KO mice (Fig. 4).

### CysLTR1 depletion drives reduced pulmonary glucose availability

The most important finding of this study is the significantly reduced glucose (and galactose) levels in the BALF of CysLTR1 KO mice, confirming a previously reported association between CysLTRs and pulmonary glucose homeostasis (Filgueiras et al., 2015; Mothe-Satney et al., 2012). Mechanistically, CysLTR1 has been implicated in the regulation of insulin secretion and glucose handling. Guo et al. (2018) reported reduction in glucose-stimulated insulin secretion in MIN6 cells, a mouse pancreatic beta-cell line, via CysLTR1. In the absence of CysLTR1, insulin secretion is improved, resulting in improved systemic glucose clearance and glucose uptake into insulin-dependent tissues, including skeletal muscle, adipose tissue and the heart. This phenomenon results in reduced glucose availability to other organs that take up glucose via non-insulin-dependent pathways, including the lung, and hence results in reduced glucose levels in the BALF of CysLTR1 KO mice as seen in this study. This observation provides important insight into the role of CysLTR1 in regulating the metabolic microenvironment of the lung, particularly under inflammatory conditions. CysLTR1, a receptor for the leukotrienes LTC₄, LTD₄, LTE₄ and LTB₄ (Emala, 2018), plays a critical role in mediating inflammatory responses in the lung (Figueroa et al., 2001; Zhu et al., 2012). The absence of CysLTR1 results in a disruption of the normal leukotriene-mediated signaling cascade, and subsequently a reduced inflammatory cell response in the airways. Activation of immune cells such as M1 macrophages, neutrophils, and T lymphocytes, induces metabolic reprogramming. These cells become highly dependent on glycolysis during inflammation and as a result, they consume large amounts of glucose (Pająk et al., 2024; Soto-Heredero et al., 2020). The observed reduction in glucose levels in the BALF of CysLTR1 KO mice may therefore reflect a reduced basal pulmonary inflammatory response, resulting from the disruption of leukotriene-mediated signalling.

### Downstream effects of CysLTR1 depletion on glycolysis-linked pathways

Although glucose is a fundamental energy source and a key metabolite in glycolysis, it also serves as a substrate for the pentose phosphate pathway (PPP), hexosamine pathway, and tricarboxylic acid (TCA) cycle. This is consistent with the reduced levels of glucose in this study which led to downstream metabolic perturbations. Carbohydrate-related metabolite intermediates including myo-inositol, D-chiro-inositol, glyceric acid, glucosamine, gluconic acid, and sedoheptulose, all of which are directly or indirectly linked to glucose metabolism were significantly reduced (Fig. 4).

Myo-inositol and D-chiro-inositol are isomeric forms of inositol, a sugar alcohol involved in cellular growth and insulin functionality (DiNicolantonio & O’Keefe, 2022). Both metabolites are synthesized from glucose-6-phosphate, as shown in Fig. 4. In the absence of an adequate glucose supply, the availability of glucose-6-phosphate is limited, leading to reduced inositol biosynthesis (Lepore et al., 2021). Since myo-inositol is the precursor of D-chiro-inositol, a further reduction in the latter supports this finding. The depletion of these intermediates not only indicates impaired glucose metabolism (Lepore et al., 2021) but also a reduced insulin signal transduction (DiNicolantonio & O’Keefe, 2022) and an altered membrane phospholipid composition, particularly in tissues such as the lung and liver (Suliman et al., 2022). Subsequently, the roles of these metabolites in type 2 diabetes mellitus has been widely investigated. Myo-inositol has been reported to inhibit glucose absorption in the intestine and promote muscle glucose uptake in rats, whereas clinical trials have demonstrated that both myo-inositol and D-chiro-inositol possess insulin-mimetic properties and the ability to improve insulin sensitivity in metabolic conditions associated with insulin resistance in humans (Chukwuma et al., 2016; Jeon et al., 2016).

Glucosamine is a precursor for glycosaminoglycans and glycoprotein synthesis, macromolecules that play crucial roles in various biological processes, including cell signaling, tissue development, and disease progression (Boullanger et al., 1990; Linhardt & Toida, 2004; Smock & Meijers, 2018; Tian & Zhang, 2013). Since protein glycosylation is critical for mucosal barrier integrity and immune function in the lung, reduced glucosamine availability in the KO group may adversely affect pulmonary defense mechanisms.

### Suppression of the pentose phosphate pathway and nucleotide metabolism

The PPP functions by synthesizing nucleotide precursors, including ribose-5-phosphate (Fig. 4), and maintaining a balance between NADP⁺ and NADPH (Stincone et al., 2015; TeSlaa et al., 2023). Since inflammatory cells rely on the PPP to support rapid proliferation and anabolic metabolism, reduced inflammation and glucose supply to the PPP results in diminished PPP flux, which is consistent with the reduction in the levels of some PPP-associated metabolites, such as D-xylose, gluconic acid and sedoheptulose in the CysLTR1 KO mice in this study. D-xylose is metabolized through the nonoxidative branch of the PPP, where it is interconverted via intermediates such as xylulose-5-phosphate to eventually produce ribose-5-phosphate. Similarly, gluconic acid (derived from glucose) also serves as a precursor for ribose-5-phosphate (Stincone et al., 2015; TeSlaa et al., 2023). The reduced availability of glucose, D-xylose and their resulting PPP precursors limits the capacity of the PPP to generate ribose-5-phosphate, which is required for nucleotide synthesis. Sedoheptulose metabolism functions in NADPH generation, the latter of which is primarily used towards reductive biosynthesis reactions, and for ribose synthesis, for nucleotide biosynthesis (Perl et al., 2011). Although not identified by the strict multistatistical selection criteria used, the resulting decrease in nucleotide synthesis is further substantiated by the reduced levels of 1-methylinosine (Log2FC = − 1.182, effect size = 1.643) which showed a strong significance considering the Log2FC and effect size, a methylated purine nucleoside also involved in purine metabolism, in the CysLTR1 KO mice in our study.

### Metabolic compensation via amino acids and lipid/fatty acids

In response to reduced glucose availability, the lung undergoes a metabolic adaptation. Glutaric acid is a downstream product of lysine and tryptophan catabolism (Sauer et al., 2005). These amino acids are catabolized to glutaryl-CoA, which is then converted into crotonyl-CoA and eventually enters the TCA cycle (Trefely et al., 2020). The reduced level of glutaric acid indicates an altered amino acid metabolism and reduced anaplerotic input into the TCA cycle. However, it is more likely, considering also the reduction in mandelic acid (Ji et al., 2022), tricarballylic acid (TA) (Russell & Forsberg, 1986) and decan-2-ol (Riley et al., 2012), that the aforementioned glutaric acid is due to an altered intestinal microbiome (Cai et al., 2023) in the CysLTR1 KO mice. Recent studies have previously associated these metabolites to microbial activity and dysbiosis under inflammatory conditions (Cai et al., 2023; Yu et al., 2025).

Although there is as yet no direct link between CysLTR1 and the gut or intestinal microbiota, CysLTR1 belongs to the G protein–coupled receptors (GPCRs) family, which are the most abundant class of cell surface receptors. Importantly, GPCRs are known to interact with the gut microbiota, and these interactions influence a wide range of physiological processes (Aleti et al., 2023). Therefore, the observed reduction in these metabolites most likely results from changes to microbial metabolism associated with the loss of CysLTR1-mediated inflammatory signalling and this may indirectly modulate the host–microbiome interface. Various inflammatory mediators, including leukotrienes, have been reported to influence the composition and activity of microbial communities (Serezani et al., 2005).

Decreased levels of fatty acid–related metabolites, including 1-hexadecanol and butanoic acid (Fig. 4), suggest altered lipid utilization for energy availability in the lung. Given that leukotrienes are lipid mediators derived from arachidonic acid (Di Gennaro & Haeggström, 2012), and play a key role in inflammatory processes, disruption of CysLTR1-mediated signaling likely affects both inflammatory lipid pathways and broader fatty acid metabolism (Zhang et al., 2023).

In the current investigation we used WT and KO mice under baseline conditions, describing the baseline metabolic consequences of its genetic deletion. This study serves as a basis to better understand the role of CysLTR1 under baseline conditions, and the understanding of this as a foundation to better elucidate the metabolic regulatory role of CysLTR1 under stress or various disease states, as a future objective.

### Study limitation

Despite the novel findings reported in this study, we acknowledge the limitation of a relatively small experimental sample size. Furthermore, although cysteinyl leukotriene pathways have been linked to systemic metabolic regulation in other contexts (Liu et al., 2025; Chwieśko-Minarowska et al., 2012), the present study did not assess parameters such as systemic insulin levels, whole-body glucose clearance, or glucose tolerance directly. No studies to date have investigated the role of a CysLTR1 knockout on the pulmonary metabolome in mice by analysing bronchoalveolar lavage fluid using untargeted metabolomics, making this study unique and its findings extremely valuable in the context of lung functionality and possibly later also pulmonary disease. Since this study was holistic and exploratory in nature, we subsequently identified several new metabolites associated directly with CysLTR1 in the lung using BALF, which not only provides numerous new biochemical hypotheses to direct future studies but also supports existing hypotheses or conclusions made by other groups.

## Conclusion

CysLTR1 plays a key role in a variety of metabolic pathways necessary for maintaining lung homeostasis, the most significant of which is glucose uptake and metabolism, with consequential changes to lipid, PPP, nucleotide and microbiome metabolism. These observed changes underscore the central role of CysLT1 in insulin signaling and the inflammatory response, as suggested by previous studies (Guo et al., 2018).

CysLTR1 expression is reportedly increased in the airway mucosa of asthma patients, particularly during exacerbations (Zhu et al., 2012), and an important regulator of mucus secretion, eosinophil recruitment, and airway inflammation, which are all hallmarks of asthma. This study serves as a basis towards a better understanding of the role of CysLTR1 in the lung, and many such studies to follow could be used towards inflammation related lung disease prevention or improved therapeutic strategies for such.

## Supplementary Information

Below is the link to the electronic supplementary material.

Supplementary Material 1

## Data Availability

Data are available on request from the corresponding author.

## Abbreviations

BALF: Bronchoalveolar lavage fluid
BSTFA: O-bis (trimethylsilyl) trifluoroacetamide
CysLTR1: Cysteinyl leukotriene receptor 1
Cysltr1−/−: CysLTR1-deficient
Cysltr1−/+: Heterozygous
CysLTs: Cysteinyl leukotrienes
GC-TOFMS: Gas chromatography-time of flight mass spectrometry
IL-6: Interleukin 6
KO: Knockout
LTB4: Leukotriene B4
NF-κB: Nuclear factor kappa B
NIST: National Institute of Standards and Technology
PCA: Principal component analysis
PPP: Pentose phosphate pathway
QT: Quality control
TA: Tricarballylic acid
TCA: Tricarboxylic acid
TMCS: Trimethyl chlorosilane
TMS: Trimethylsilyl
TNF-α: Tumor necrosis factor-α
VIP: Variable of importance in projection
WT: Wild-type
