# Supplementary material for: The functionality of the cysteinyl leukotriene receptor 1 (CysLTR1) in the lung by metabolomics analysis of bronchoalveolar lavage fluid
Source: Metabolomics. 2026 Jul 8;22(4):121. doi: 10.1007/s11306-026-02491-9 (PMC13346134; doi:10.1007/s11306-026-02491-9)
Supplement: Supplementary file 1 — Supplementary Material 1 [file 11306_2026_2491_MOESM1_ESM.pptx]

## Slide 1
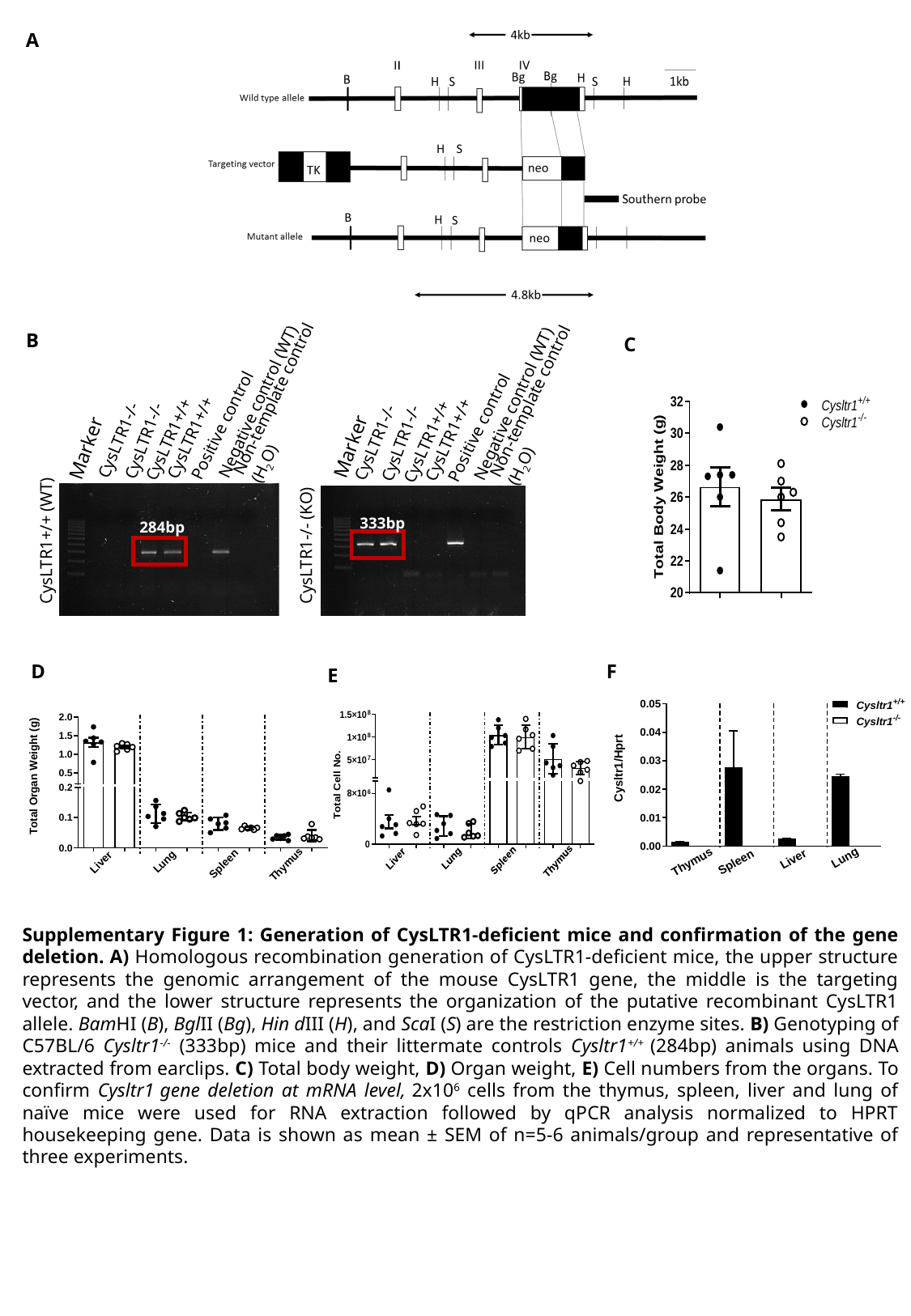

A
Non-template control (H2O)
Non-template control (H2O)
Positive control
Negative control (WT)
Positive control
Negative control (WT)
CysLTR1+/+
CysLTR1+/+
Marker
CysLTR1+/+
Marker
CysLTR1+/+
CysLTR1-/-
CysLTR1-/-
CysLTR1-/-
CysLTR1-/-
CysLTR1+/+ (WT)
CysLTR1-/- (KO)
B
333bp
284bp
C
D
F
E
Supplementary Figure 1: Generation of CysLTR1-deficient mice and confirmation of the gene deletion. A) Homologous recombination generation of CysLTR1-deficient mice, the upper structure represents the genomic arrangement of the mouse CysLTR1 gene, the middle is the targeting vector, and the lower structure represents the organization of the putative recombinant CysLTR1 allele. BamHI (B), BglII (Bg), Hin dIII (H), and ScaI (S) are the restriction enzyme sites. B) Genotyping of C57BL/6 Cysltr1-/- (333bp) mice and their littermate controls Cysltr1+/+ (284bp) animals using DNA extracted from earclips. C) Total body weight, D) Organ weight, E) Cell numbers from the organs. To confirm Cysltr1 gene deletion at mRNA level, 2x10^6^ cells from the thymus, spleen, liver and lung of naïve mice were used for RNA extraction followed by qPCR analysis normalized to HPRT housekeeping gene. Data is shown as mean ± SEM of n=5-6 animals/group and representative of three experiments.
